# The paranormal health beliefs scale: an evaluation using cognitive interviewing

**DOI:** 10.3389/fpsyg.2024.1306372

**Published:** 2024-01-29

**Authors:** Andrew Denovan, Neil Dagnall, Kenneth Graham Drinkwater

**Affiliations:** Manchester Metropolitan University, Manchester, United Kingdom

**Keywords:** cognitive interviewing, illusory thinking, paranormal health beliefs, paranormal health beliefs scale, questionnaire scrutiny

## Abstract

Paranormal health beliefs denote the inclination to endorse illusory supernatural notions about well-being and treatment. These ideations are important since they potentially influence perceptions of health and allied behaviors. Noting this, researchers in Italy developed and verified the Paranormal Health Beliefs Scale (PHBS). Despite initial promising outcomes, the construct and measurement properties of the PHBS have remained under investigated. This is likely due to the fact that the instrument draws heavily on traditional Italian social, political, and religious influences and is overly culturally specific. Hence, items do not generalize well across populations and nationalities. Acknowledging these factors, this study used cognitive interviewing (think aloud protocol and concurrent probing) to assess the suitability of the PHBS for general use. Concurrently, the intention was to identify necessary modifications that would enhance scale performance. Fourteen interviewees (eight males and six females), evenly distributed across two rounds, participated. Round 1 focused on comprehension and perception of the PHBS. Cognitive interviews identified issues with culturally particular content/points of reference, phraseology, and wording. To address these a modified version of the PHBS was produced. Round 2 then examined the effectiveness of changes. Analysis revealed fewer concerns, although difficulties with ambiguity, complex terminology, and response scale appropriateness persisted. Overall, interviews indicated that a focus on illusory (rather than paranormal) health beliefs would improve scale utility. Methodologically, cognitive interviewing provided invaluable insights into the effectiveness of the PHBS and identified ways in which researchers could adapt the instrument for use with other cultures.

## Introduction

In the 1950s, the US Public Health Service developed the Health Belief Model (HBM) (Hochbaum et al., 1952). Researchers used the HBM to explain failures to engage with disease screening and prevention strategies. Later, theorists applied the HBM to symptom response and compliance with medical interventions (Skinner et al., 2015). The HBM derives from the notion that beliefs about illness/disease and/or threat combined with perceived efficacy of health behaviors predict probability of engagement. Hence, health behavior is determined by subjective assessment of disease/illness severity alongside imagined benefits/barriers to initiating allied responses (Etheridge et al., 2023).

The HBM originated from two related behavioral components, intention to avoid illness and/or recover from sickness, and faith that a health-related action prevents/cures illness (Champion and Skinner, 2008). These components reflect value-expectancy concepts. The former represents the value placed upon evading illnesses and recovery, whereas the latter reflects the belief that specific health actions stop or ameliorate ill health. This approach draws heavily on the cognitive notion that the subjective hypotheses and expectations held by individuals predict actions. Thus, health behavior reflects the perceived value of an outcome (subjective) combined with the personally assigned probability (expectation) that a particular activity will achieve a specific outcome (Skinner et al., 2015).

Within the HBM there are six elements, four original core features associated with perceptions (susceptibility, severity, benefits, and barriers) and two additional features, appended as the model evolved (cue to action and self-efficacy) (Rosenstock et al., 1988). Susceptibility refers to beliefs about the probability of risk of contracting a condition/disease. Severity denotes judgment of condition and sequalae seriousness. Benefits describes believed efficacy of recommended/available actions to reduce risk. Barriers designate assessments of the evident and psychological costs of action, incorporating cost/benefit analyses. Cues to action specifies the required stimuli to activate willingness to adopt a health behavior (e.g., internal symptoms, advice from health professionals). Self-efficacy represents an individual’s confidence in their ability to initiate appropriate action. For further detail see Champion and Skinner (2008), who define these features in detail, provide application examples, and outline relationships between features.

The HBM is important because it explains how subjective evaluations of illness/disease affect initiation of preventative/treatment-based actions (Laranjo, 2016). Acknowledging this and recognizing the prevalence of paranormal beliefs in contemporary Western societies (see Dagnall et al., 2016, 2022), Donizzetti and Petrillo (2017) developed the Paranormal Health Beliefs Scale (PHBS). Although, levels of belief vary as a function of survey questions and respondent types, reported incidence is typically high (i.e., approximately 50% of the sampled population; Marks, 2021) (Williams et al., 2022). Combining personal judgments about well-being with supernatural credence, the PHBS delimits paranormal health beliefs (PHBs) as views that exceed the limits of what is considered physically possible according to prevailing scientific assumptions. Correspondingly, the instrument assesses respondents’ inclination to endorse supernatural-based notions about well-being and treatment.

PHBs are important because they can help individuals to cope with health-related concerns. However, beliefs become maladaptive when supernatural ideations undermine science-informed approaches (Farias et al., 2013; Dagnall et al., 2019). In such circumstances, PHBs can negatively influence attitudes, outcome expectancies, and behaviors. For instance, disrupt/prevent engagement with conventional medical treatment (e.g., encourage individuals to avoid undertaking health procedures on certain dates such as Friday 13th) and/or motivate the use of specious interventions/cures (e.g., potions).

From this perspective, PHBs represent illusory ideations about mental and physical well-being that hinder established diagnostic and therapeutic processes and/or impair involvement with and adherence to conventional treatments (Capone, 2016). This interpretation aligns with the conceptualization of paranormal experiences and beliefs as a manifestation of non-clinical delusional thinking (Irwin et al., 2012a,b; Drinkwater et al., 2021). Commensurate with this supposition, Donizzetti and Petrillo (2017) operationalized the relationship between paranormal beliefs and health in terms of self-serving illusions (Yarritu et al., 2015). Whereby, belief structures well-being-related ideations, so that they are meaningful and consistent with the individual’s worldview. This provides ontological security (i.e., the perception that personal experiences possess order and continuity), emotional protection (i.e., shield individuals from the uncertainties of life), and reassurance (i.e., sense of control and meaning) (Irwin, 1993, 2009).

In this context, studies have reported that ill-founded notions about well-being were associated with poorer engagement and maintenance of treatment/therapy. Illustratively, irrational health beliefs predicted poorer adherence to rehabilitative care in sufferers of cardiovascular diseases and diabetes (Anderson and Emery, 2014). Correspondingly, studies report that paranormal beliefs predict faith in alternative and complementary medicine (Pettersen and Olsen, 2007; Van den Bulck and Custers, 2010). These represent healthcare approaches that have developed outside evidence-based frameworks (Li et al., 2018). Alternative medicine is a replacement to evidence-based medicine, whereas complementary medicine is used in addition to conventional approaches. Allied to these findings, other investigators have observed that paranormal belief-related constructs influence health behaviors. For instance, religious and fatalistic beliefs are associated with lower engagement with healthcare (Gall et al., 2005; Franklin et al., 2007).

Commensurate with this perspective, investigators in the area of mental health have developed scales to assess relationships between delusional thinking and psychological wellbeing. A frequently cited example is the Magical Ideation Scale (Eckblad and Chapman, 1983), which measures belief in unconventional forms of causation. Specifically, paranormal credence (e.g., superstition, reincarnation and telepathy), religious beliefs, and general magical thinking, including psychotic symptoms such as odd and unusual beliefs and delusions of reference. Noting this, Kingdon et al. (2012) developed the Illusory Beliefs Inventory (IBI) for use with non-clinical populations. The IBI comprises three factors: magical beliefs (i.e., general faith in unseen/unknown forces), spirituality (i.e., endorsement of spiritual/higher powers), and internal state and thought action fusion (i.e., focus on intrapsychic activity and tendency to believe that intrusive thought increases the likelihood of event occurrence and/or is the moral equivalent of action). Concomitantly, researchers have also developed scales to appraise superstitiousness. Illustrations include the Lucky Beliefs and Behaviors Scales (Frost et al., 1993), which evaluate the tendency to engage with superstitious credence and actions to promote good fortune, and the Superstitiousness Questionnaire (Zebb and Moore, 2003), a general measure of endorsement of common Western superstitions. Collectively, scales such as these have provided important insights into a range of mental health-related conditions and syndromes (e.g., compulsivity and psychological distress).

Cognizant of the influence of illusory health beliefs and the lack of specific measurement appropriate instruments, Petrillo and Donizzetti (2012) and Donizzetti and Petrillo (2017) developed and verified the PHBS. Prior to the PHBS, assessment of PHBs was restricted to small numbers of items subsumed within general supernatural measures. For example, the Supernaturalism Scale (Randall and Desrosiers, 1980) contains items referencing faith healing and the limitations of modern medicine, included within a global Supernaturalism factor, and Nixon’s Superstitions Scale (Nixon, 1925) refers to the healing powers of paranormal forces incorporated within a global Superstitiousness dimension. The most commonly used measures, the Revised Paranormal Beliefs Scale (Tobacyk, 2004) and the Australian Sheep Goat Scale (Thalbourne, 1995), make no explicit reference to health (see Drinkwater et al., 2017b, 2018).

To develop the PHBS, Petrillo and Donizzetti (2012) generated a breadth of content-related items. These were used to assess adolescents’ illusory beliefs about health. The item pool was administered to 1,469 adolescents, and responses subjected to exploratory and confirmatory factor analysis. Analyses identified a multidimensional structure comprising five belief types: Religious (elements of faith allied to health protection/recovery), Superstitious (practices that ward off health threats), Extraordinary Events (unknown entities/events/force that influence health, e.g., universal forces), Parapsychological (mental energies, which affect health), and Pseudo-scientific (health threats caused by specific deviant or marginal social groups). Satisfactory psychometric properties (i.e., internal reliability and discriminant validity) were observed. Petrillo and Donizzetti (2012) accordingly concluded that the emergent 31-item PHBS was an effective tool for evaluating illusory beliefs related to adolescents’ health.

In a subsequent validation study, Donizzetti and Petrillo (2017) administered the PHBS to 643 participants in a university-based sample. Snowball sampling produced a fairly equal gender balance and a range of ages (i.e., 18 years, 48.1%; 19–30 years, 22.1%; 31–60 years, 19.3; and 61–80 years, 10.6%). Analysis confirmed the dimensions identified in the pilot study and, via correlations with locus of control and self-efficacy, further demonstrated convergent and discriminant validity. Overall, results confirmed those from the pilot study, indicating that researchers could use the instrument to identify PHBs across the lifecycle. Notwithstanding these promising outcomes there has been only limited subsequent research with the PHBS (e.g., Rosa, 2018). Consequently, despite being an important research tool for exploring the impact of illusory health beliefs, the measurement properties of the PHBS remain under investigated. Particularly, further research is required to ensure the appropriateness of PHBS contents for other samples.

Hence, the present study used cognitive interviewing to assess the validity of the PHBS for use with English-speaking general populations. Specifically, reviewed item clarity and relevance (Peterson et al., 2017). Cognitive interviewing is a combination of cognitive psychology and survey methodology, involving asking respondents to think aloud as they progress through a survey. By directing respondents to verbalize their thoughts and perceptions, this process provides essential insights into respondents’ perceptions of scale content. Thus, it is a useful technique for exploring the strength and weaknesses of scale content, particularly statement wording/meaning.

This process ensures that items are interpreted consistently and adequately assess construct domain (Ryan et al., 2012). Moreover, cognitive interviewing identifies response issues (Drennan, 2003). Common problems are lexical (e.g., inexact wording producing misunderstanding), inclusion/exclusion (e.g., inappropriate generalization/restriction), temporal (e.g., respondents unclear about the time period being assessed), logical (e.g., respondents concurrently responding to different question elements), and computational (difficulties not subsumed within the other categories such as long-term memory errors and those requiring complex estimation) (Conrad and Blair, 1996).

A further strength of cognitive interviewing is its ability to assess items in terms of cognitive operations (i.e., comprehension, recall, judgment, and response) (Tourangeau, 1984). Although the order of operations varies, accurate answers depend on participants’ ability to understand what questions and statements mean, retrieve pertinent information or knowledge, make a judgment based on recall, and select a suitable response (Ryan et al., 2012). Factors that impair operations result in misalignment between participant interpretation and developer intentions. Thus, cognitive interviewing identifies ways in which researchers can improve items to enhance the quality of participants’ responses (Peterson et al., 2017).

## Method

### Participants

The sample comprised 14 participants (evenly distributed between Round 1 and Round 2). Mean age was 39 (range 24–64). There were eight males (*Mean* age = 44, range 24–64), and six females (*Mean* age = 34, range 25–62). The sample was purposive, selection of participants being informed by their knowledge of relevant psychological concepts. Accordingly, all participants possessed theoretical understanding of paranormal phenomena and/or health-related issues obtained via study/research of relevant issues at undergraduate level and beyond. To take part, participants were at least 18 years of age, did not suffer from any diagnosed psychological illness, resided in the UK, and were classed as a British citizen. Table 1 contains participant characteristics.

**Table 1:** Participant characteristics.

Participant	Gender	Age	Educational level	Cognitive interview
1	Male	64	Postgraduate	Round 1
2	Male	41	Postgraduate	Round 1
3	Female	62	Undergraduate	Round 1
4	Male	54	Postgraduate	Round 1
5	Male	53	Postgraduate	Round 1
6	Female	26	Undergraduate	Round 1
7	Male	35	Postgraduate	Round 1
8	Male	38	Postgraduate	Round 2
9	Female	25	Undergraduate	Round 2
10	Female	25	Undergraduate	Round 2
11	Male	24	Undergraduate	Round 2
12	Female	29	Postgraduate	Round 2
13	Male	43	Further Education	Round 2
14	Female	37	Undergraduate	Round 2

### Measure

#### The paranormal health beliefs scale

The paranormal health beliefs scale (PHBS) (Petrillo and Donizzetti, 2012) is a 31-item instrument, which assesses the tendency to endorse supernatural notions about well-being. Items appear as statements (e.g., ‘Illness can be overcome by force of mind’) and respondents indicate their level of agreement via a five-point Likert response scale (1 = Strongly Disagree to 5 = Strongly Agree). The PHBS has demonstrated acceptable reliability, with reported estimates from 0.65 to 0.91, and factorial validity (Donizzetti and Petrillo, 2017).

### Procedure

Potential participants were given an information sheet, those providing informed consent progressed to interview. The lead researcher, who was trained in cognitive interviewing, asked participants to complete the PHBS, whilst concurrently outlining their thoughts. This think aloud protocol was accompanied by concurrent probing (i.e., questioned during completion, e.g., “can you say what you think the question is asking?”). The interview protocol allowed the researcher to identify statements within the PHBS that were ambiguous, poorly worded, and/or misleading.

Testing was conducted in two rounds, the first assessed the original PHBS and the second evaluated the effectiveness of modifications. Each round terminated when saturation was achieved (i.e., no new issues were evident). In both rounds, participants were also asked to recommend how unclear items could be phrased more appropriately. In the second round, the research asked participants to suggest additional statements. Interviews lasted approximately 1 h. After taking part, all participants were debriefed. Ethical approval was granted by the Manchester Metropolitan University Ethics Committee (EthOS ID #52313).

## Results

### Analysis

Data were coded and analyzed using Tourangeau’s framework (1984, modified by Willis, 1999). This considers four significant cognitive aspects of question answering. Explicitly, ‘comprehension’ (understanding), ‘retrieval’ (how information is accessed from memory), ‘decision’ (deriving answers), and ‘response’ (the extent to which responses occur without error/obstruction).

#### Round 1 (evaluation)

##### Comprehension

Three main forms of comprehension issues (i.e., social/cultural equivalence of beliefs/behaviors, ambiguous language, and conceptually complex items) were observed (see Table 2). Typically, participants perceived items referring to Catholicism and Italian culture as too specific (e.g., ‘faith in the saints heals many diseases’). This was true for approximately a third of the items. In these cases, participants were able to suggest appropriate alternatives (i.e., for UK and US samples use Friday13th rather than 17th as an unlucky date) or general statements (i.e., ‘some dates are associated with bad luck’). Nonetheless, participants were typically able to identify the underlying construct (e.g., superstition).

**Table 2:** Summary of types of problems identified during cognitive interviews (Round 1).

	Cognitive processing problem			
PHBS item	Comprehension^a^	Retrieval^b^	Decision^c^	Response^d^
1	Ambiguous language – ‘force of mind’	–	Uncertainty of meaning impacted decision	Chose positive responses on the belief it referred to something else (willpower)
2	Cultural equivalence of ‘Friday 17th’	Lack of direct experience of ‘surgical interventions’	Uncertainty impacted decision	–
3	Ambiguous language – ‘some persons,’ ‘force of mind’	–	Uncertainty impacted decision	Chose positive responses on the belief it referred to something else (willpower)
4	Cultural equivalence of ‘saints’	–	Uncertainty impacted decision	Often chose ‘do not know’ due to ambiguity
5	Conceptually complex – ‘the soul’	–	–	–
6	–	–	–	–
7	Cultural equivalence of ‘saints’	–	Uncertainty impacted decision	Often chose ‘do not know’ due to ambiguity
8	Cultural equivalence – ‘kissing relic or statue of saint’	–	Uncertainty impacted decision	Often chose ‘do not know’ due to ambiguity
9	–	Outlandish and unable to relate – ‘extra-terrestrial entities’	–	Often chose ‘do not know’ due to ambiguity
10	Ambiguous language – ‘some people,’ ‘power’	–	–	–
11	Cultural equivalence – receiving object touched by a saint	–	Uncertainty impacted decision	Often chose ‘do not know’ due to ambiguity
12	–	Conflated in relation to health	–	–
13	Cultural equivalence of ‘touching iron’	–	Uncertainty impacted decision	Often chose ‘do not know’ due to ambiguity
14	–	–	–	–
	Cognitive Processing Problem			
15	Ambiguous language – ‘cosmic energy’	–	–	–
16	–	–	–	–
17	–	Outlandish and unable to relate – ‘contact with alien species’; conflated in relation to health	–	–
18	Cultural equivalence of ‘evil eye’	–	–	–
19	Ambiguous language – ‘mental defense strategies’	–	–	Chose positive responses on the belief it referred to something else (psychological defenses)
20	–	–	–	–
21	Convoluted language	–	–	–
22	–	–	Inappropriate item; unaligned with others	–
23	Convoluted language	–	–	–
24	–	–	–	–
25	–	–	–	–
26	–	–	Inappropriate item; unaligned with others	–
27	Cultural equivalence of ‘amulet’	–	–	–
28	Ambiguous language – ‘mental energies’	–	–	–
29	–	–	Inappropriate item; unaligned with others	–
30	Cultural equivalence – ‘touching pregnant woman’s tummy’	–	Uncertainty impacted decision	Often chose ‘do not know’ due to ambiguity
31	–	–	Inappropriate item; unaligned with others	–

^a^Understanding the question; ^b^retrieving necessary information from memory; ^c^error introduced when deciding on the response; ^d^response scale adequacy.

Ambiguous language undermined meaning in approximately a quarter of items. This resulted in conflation between paranormal and psychological powers/forces. For instance, ‘force of mind’ was often interpreted as willpower/resilience and ‘mental defense strategies’ were perceived as protection mechanisms. Moreover, it was not clear what was meant by the ‘soul’.

Some items (e.g., 23 and 29) contained convoluted wording. This is highlighted by item 23, which fails to adequately define ‘health conditions’ and concomitantly presents a vague, overly long notion of spiritual separation. Subsequently, it is unclear whether the outlined phenomena refer to an out-of-body (OBE) or near-death experience (NDE). While these occurrences often overlap, they are not mutually inclusive. Consequently, from a health perspective OBEs and NDEs are associated with different health states. Explicitly, parapsychological literature designates that OBEs typically occur during normal dissociative states, whereas NDEs happen during acute medical emergencies.

##### Retrieval

Although participants were often able to access topic relevant information from memory, this process was obfuscated when items were culturally specific. In such instances, reframing was required prior to response. This involved reconceptualizing statements in a personally relevant manner (e.g., item 7, where respondents believed that faith in God rather than saints facilitated health). When items referenced highly improbable phenomena (i.e., extra-terrestrial induced health issues/disease) and low frequency events (i.e., eclipses), participants experienced difficulties finding appropriate points of personal relevance.

##### Decision

In addition to the issues outlined above, participants’ judgments of item adequacy were affected by sensitive content. Explicitly, pseudo-scientific statements (e.g., items 22, race; 29, homosexuals; and 31, immigrants) that refer to health threats caused by marginal social groups. Participants expressed discomfort with these items because they were politically and socially insensitive. Moreover, there was a consensus that since these items implied the presence of prejudicial rather than paranormal views they should be removed.

##### Response

Participants experienced difficulties when they responded to hard to comprehend items. Some failed to respond, whereas others provided approximations and uncertain answers. Additionally, to provide context and frame expectations, participants generally recommended amending the scale instructions to emphasize that items are assessing the relationship between paranormal beliefs and health. Finally, because statements were positively worded some participants reported that they found responding repetitive.

#### Round 2 (modification)

##### Summary of changes

Round 2 implemented PHB modifications identified in Round 1 (see Table 3). To facilitate comprehension, culturally specific references were amended, and ambiguous phrases reworded. Relevance to UK and US participants was increased by appending superstition items with more general, familiar content (e.g., ‘touching wood wards off threats to health’). Moreover, items were incorporated that included well-known health misperceptions, such as ‘cracking knuckles’ and its link to arthritis, and ‘feeding a cold and starving a fever’. To improve accessibility and reduce participant discomfort, politically and socially insensitive items were removed. To reduce potential response bias, negatively keyed items were added to the scale. Also, instructions specifying the purpose of the PHB were appended. These stated that forces or powers denoted paranormal/supernatural phenomena and contextualized the statements to follow. Finally, items were more closely aligned to established paranormal domains as identified by established measurement instruments such as the Australian-Sheep Goat Scale and the Revised Paranormal Belief Scale (extrasensory perception, psychokinesis, superstition, etc.) and less focus was placed on Catholicism particularly and religious belief generally.

**Table 3:** Modified versus original questions.

Original item^a^	Modified item	Final wording^b^
1. Illness can be overcome by force of mind	Illness can be overcome by mental forces	Illness can be overcome by *psychic forces*
2. Preferably avid surgical interventions on Friday 17th	Preferably avoid visits to the doctor on certain dates (e.g., Friday 13th)	*I prefer* to avoid *medical appointments (*e.g.*, doctor, dentist)* on certain dates, *for instance* Friday 13th
3. Some persons have the power to influence health through force of mind	People can influence health through mental forces	People can influence health through *psychic forces*
5. The soul exerts an influence on health	The soul or spirit can exert an influence on health	The soul or spirit can *influence health*
6. Reading horoscopes is important to maintain a good state of health	Unchanged	*Horoscopes can provide important information about health*
7. Faith in the saints heals many diseases	Religious faith heals many diseases	Unchanged
13. Touching iron wards off threats to health	Touching wood wards off threats to health	*Superstitions, such as saying ‘touch wood’ or actually touching wood,* ward off threats to health
14. Holy water protects the health of the person who drinks it	Holy water protects the health of people	Holy water protects *against illness and disease*
16. Cases of healing due to strength of faith do exist	Cases of healing due to strength of religious faith do exist	Unchanged
18. The evil eye may influence the state of health of a person	Curses may influence the state of health of a person	Curses *may cause illness*
20. Guardian angels keep away illnesses	Guardian angels or other spiritual forces can protect me against illness	Guardian angels or other spiritual forces can protect against illness
21. States of malaise can facilitate the release of the spirit from the body to enter into another body or another place	Unchanged	*States of illness can facilitate the separation of the spirit from the body*
23. In certain health conditions it is possible to feel that one’s own spirit is floating out of one’s own body or to perceive one’s own body from an external position	Unchanged	Unchanged
24. Breaking glass or a mirror does not bode well for health	Unchanged	Unchanged
25. Health is in the hands of God	Unchanged	Unchanged
27. Wearing an amulet may help to keep one healthy	Wearing an amulet or a lucky charm helps to keep one healthy	Unchanged
28. Changes in health conditions (such as increase in body temperature or quickening of heartbeat) may be provoked by mental energies	Mental forces can provoke changes in health conditions (such as an increase in body temperature or a quickening of the heartbeat)	*Psychic forces* can provoke changes in health conditions (such as an increase in body temperature or a quickening of the heartbeat)

^a^Items 4, 8, 9, 10, 11, 12, 15, 17, 19, 22, 26, 29, 30, 31 were deleted following Round 1; ^b^changes to wording during Round 2 indicated in italics.

##### Comprehension

Round 2 (vs. Round 1) found fewer comprehension issues (i.e., five items). The problem with these items were ambiguity and use of complex terminology. Illustratively, item 21 failed to adequately delineate ‘states of malaise’ and combined the phrase with the imprecise notion of spiritual separation. Consequently, it was unclear whether the item referred to an out-of-body or religious experience. Following Round 2, items that participants defined as unclear were re-evaluated. In the case of item 21, ‘malaise’ was changed to illness, and the item was appropriately truncated. Moreover, despite changing ‘force of mind’ to ‘mental forces’ participants still misconstrued the phrase as psychological (willpower) rather than supernatural. Participant feedback indicated that subsequent item iterations should use ‘psychic forces’ as this was considered a more exact term to denote paranormal phenomena.

##### Retrieval

As with Round 1, experience of phenomena influenced retrieval. For instance, a participant who reported an OBE identified strongly with the descriptions provided. Similarly, a participant who experienced visions of becoming ill prior to sickness referred to allied items as highly appropriate.

##### Decision

Item ambiguity impaired responses (as in Round 1). For example, the conflation between psychological and paranormal processes remained evident in a subset of items. Additionally, the item ‘touching wood wards off threats to health’ was not wholly reflective of the superstition since some participants reported that it is ‘say’ or ‘touch’. The item was rephrased accordingly. Nonetheless, it was clear from the interviews that the amendments had improved clarity for general, English-speaking samples. Furthermore, there was uncertainty whether superstition items (e.g., ‘I believe that eating an apple a day will keep the doctor away’) represented notions of luck or were merely a proverb. Finally, participants struggled to respond to the item ‘hunches about becoming ill come true and are not just ‘coincidences.” This is because hunches can refer to paranormal phenomena such as signs and precognition and/or cognitions guided by emotional responses.

##### Response

The instructions added to the beginning of the PHBS failed to resolve the ambiguity between psychological and paranormal phenomena. They also restricted responses to paranormal rather than illusory beliefs. Therefore, the instructions were deleted. Participants found that the response category of ‘Do not know’ in the middle of the response scale was an unclear choice and one that did not engender conviction. Accordingly, the response option ‘Neither agree nor disagree’ was added.

##### Face validity

Participant feedback indicated that the PHBS was assessing illusory rather than paranormal health beliefs. It was also recommended that pseudoscientific practices allied to health should be included. Accordingly, the researchers will add pseudoscience items to the next scale iteration.

## Discussion

Although psychometrically validated, due an emphasis on culturally specific and religious material, the PHBS’s utility outside of its native country is limited. For instance, the PHBS delineates Friday 17th as an unlucky day. In countries such as the UK and USA there are no negative connotations linked to the number 17, instead it is Friday 13th. Furthermore, it refers to ‘the evil eye’ as a negative influence on health. This notion is subject to geographical variation. Belief in the evil eye is prominent in locations such as the Mediterranean and Balkans and less influential in other regions. In terms of religious content, the PHBS draws on Roman Catholic symbols and iconography (e.g., saints, relics, and holy water). These do not have the same significance and connotations in highly secular societies, or those where other religions prevail.

The specificity of these items is also problematic because non-endorsement does not necessarily indicate absence of belief. With reference to lucky/unlucky days there are alternative interpretations. Firstly, a respondent could believe Friday 17th is unlucky and avoid certain activities but their conviction is not sufficient to prevent them from engaging with important life events such as surgery and work. Secondly, a respondent could provide a disagree answer because they actually believe that Friday 17th is a lucky, rather than unlucky, day. Thirdly, while the respondent does not consider Friday 17th unlucky, they may dislike other dates for superstitious reasons. These illustrations demonstrate that paranormal belief items are more effective when they assess general ideation rather than particular instances. Using the example of superstition and dates, this would involve asking participants the extent they believed that certain dates such as Friday 17th are associated with good and/or bad luck (see Drinkwater, 2017).

Independent of potential cultural bias and specificity, a heavy reliance on religious content is problematic because some societies, while demonstrating relatively high levels of paranormal belief, are more secular. Noting the conceptual difficulty of distinguishing between religious and paranormal beliefs, Baker et al. (2016) proposed the theory of bounded affinity. This draws on the observation that despite shared characteristics, organized religion constrains acceptable and true beliefs to a narrow subset of explanatory frames and occurrences. Accordingly, paranormal credence is best defined as acceptance of beliefs and experiences that overtly reject the tenets of science and organized religions.

A further limitation of the PHBS as a measure of paranormal, rather than illusory, beliefs is that items on occasion conflate concepts. This is true in the case of statements alluding to powers, forces, and energies. It is unclear in these instances what the paranormal power being indexed is. Presumably, it is psychokinesis or telekinesis, the psychic ability to influence matter. However, this is not unequivocally established by item wording. Accordingly, respondents could interpret the item as referring to positive thinking or even conventionally defined mind over matter approaches (psychological processes), which propose that self-control of thought can regulate feelings, situations, or events. Within the psychological literature there is support for these notions. For example, Jamieson et al. (2012) reported that participants (vs. controls) who were instructed to reappraise their arousal exhibited more adaptive cardiovascular stress responses (i.e., increased cardiac efficiency and lower vascular resistance, and decreased attentional bias). Thus, items within the PHBS should explicitly link outcomes to paranormal as opposed to natural and scientifically explainable phenomena.

Another issue with the content of the PHBS is that some items are socially awkward and touch on sensitive topics (e.g., race, homosexuality, and immigrants). These items ask whether interactions with a particular group are harmful to health without explicit reference to the supernatural. The inclusion of these items in a paranormal scale is questionable as they index social tolerance and prejudice as opposed to supernatural credence. Certainly, these items lack validity since they are assessing openness to health myths rather than paranormal beliefs.

A final problem with statements used in the PHBS is lack of precision. Examples of this are evident within the Extraordinary Events Beliefs subscale where the terms ‘states of malaise’ and ‘certain health conditions’ refer to well-being. This is likely because the PHBS was produced in Italian (see Petrillo and Donizzetti, 2012) and the translated version has yet to be refined. This does not diminish the importance of the instrument but illustrates that the PHBS requires modification for use with general English-speaking populations. Nonetheless, it remains the case that the translation process was not explained within the extant literature. This is necessary if the scale is to become more widely used within research.

Regarding domain content, the PHBS samples a relatively limited range of paranormal phenomena in comparison to construct breadth. This is best conceptualized in terms of work examining commonality between established paranormal measures. Explicitly, Dagnall et al. (2010) identified eight common factors: Hauntings, Superstition, Religious Belief, Alien Visitation, Extrasensory Perception, Psychokinesis, Astrology, and Witchcraft. While these overlap with PHBS subscales (i.e., Religious, Superstitious and Parapsychological Beliefs) and item content, there are important theoretical gaps (i.e., Hauntings, Extrasensory Perception, and Witchcraft) (see Drinkwater, 2017). Moreover, within the PHBS there is only partial and/or vague indirect reference to Alien Visitation, Psychokinesis, and Astrology. This indicates that the PHBS, as a function of its focus on particular PHBs, provides idiosyncratic coverage of the construct domain. Furthermore, Pseudo-Scientific Beliefs unless explicitly linked to supernatural causes are not necessarily paranormal. Regarding Extraordinary Events Beliefs, this subscale is restricted to extra-terrestrial entities, cosmic energy, and spirits.

In addition to concerns about PHBS breadth and content, it is important to note that PHBS validation was limited. Tests for ceiling and floor effects, item difficulty relative to sample, and assessment of measurement bias were not reported in the pilot and validation studies. Collectively, this indicates that further work is required to refine the PHBS. It is vital that this is undertaken as the PHBS is the only measure that currently assesses heath specific supernatural credence.

Currently, the PHBS assesses only the degree to which individuals endorse paranormal/illusory notions (i.e., belief). To understand the impact of beliefs it is also necessary to establish their purpose (i.e., intention). For instance, does credence reduce anxiety, act as a defense mechanism, and/or provide rationale for ignoring prevailing scientific evidence. Additionally, it is important to identify the effects of beliefs (i.e., behavior). Specifically, ascertain whether beliefs are benign or disrupt engagement with established medicine. Indeed, established health models (e.g., HBM, Theory of Planned Behavior; Ajzen, 1991) have typically formulated the role of health beliefs in this manner, focusing on belief, intention, and behavior. Integrating these features into an illusory health beliefs scale as it iteratively evolves will inform the development of a predictive model, which will potentially be able to detect individuals who are most at risk (i.e., those who engage with pseudo-scientific therapies and treatments and ignore conventional medical/scientific advice and procedures). Furthermore, data arising from this ‘holistic’ approach could inform the content, nature, and tone of health advice/education. A useful starting point in this process would be to conduct interviews with believers exploring the nature, function, and consequences of their paranormal/illusory convictions. This approach has previously revealed the profound effect that personal paranormality (i.e., beliefs, perceived experiences, and professed abilities) have on individual sense of self and perceptions of well-being (Drinkwater et al., 2013, 2017a, 2022).

Although the present study produced several important outcomes, it is important to acknowledge potential limitations regarding the implementation and application of cognitive interviewing. Firstly, the study recruited 14 participants. Although, this seems a small number, it was commensurate with several studies that have employed cognitive interviewing as a tool for evaluating scale item efficacy (see Wright et al., 2021). Moreover, there are currently no agreed adequacy principles regarding minimum sample size and composition (Beatty and Willis, 2007). This flexibility reflects the fact that cognitive interview samples are not typically intended to be representative of a population but are instead selected to represent the thoughts and issues of typical respondents (Beatty and Willis, 2007). As a norm, theorists recommend that researchers conduct cognitive interviews in rounds comprising between 5 and 15 interviews. Repeating the process facilitates iterative item amendment and addressing of issues (Willis, 2004).

Noting this, subsequent work should employ additional rounds and recruit larger more diverse samples to enhance generalizability. In the case of health behaviors this could include particular target populations. This would ensure that items were suitable for participants from groups (e.g., age, education, culture, and gender) with potentially differing perceptions and levels of understanding.

While cognitive interviewing provides a useful method for assessing the effectiveness of survey items, the approach possesses weaknesses. In addition to lack of specification of sample size required to obtain saturation, these include absence of standardized procedures, and the capacity to assess only reportable features of item response. With reference to the latter point, the process of asking participants to think aloud can in some instances interfere with response spontaneity (Conrad et al., 1999). Additionally, variations in probing across participants may influence and guide responses. In this context, as advised by Conrad et al. (1999), probing in the present study was restricted to asking respondents to amplify or elucidate unclear protocol verbalizations. Despite these issues, given the brief nature of PHBS items and the overall measure itself, cognitive interviewing provided important insights into how best to improve and develop an alternative instrument. Correspondingly, based on analysis, further iterations of the instrument will focus on *illusory* rather than *paranormal* health beliefs. This modification will increase the applicability of the measure by canvassing a broader range of health-related misperceptions.

## Data availability statement

The raw data supporting the conclusions of this article will be made available by the authors, without undue reservation.

## Ethics statement

The studies involving humans were approved by The Manchester Metropolitan University Ethics Committee (EthOS ID #52313). The studies were conducted in accordance with the local legislation and institutional requirements. The participants provided their written informed consent to participate in this study.

## Author contributions

AD: Conceptualization, Data curation, Formal analysis, Funding acquisition, Investigation, Methodology, Project administration, Resources, Validation, Visualization, Writing – original draft, Writing – review & editing. ND: Conceptualization, Visualization, Writing – original draft, Writing – review & editing. KD: Writing – review & editing.
